# Marker-Assisted Molecular Profiling, Deletion Mutant Analysis, and RNA-Seq Reveal a Disease Resistance Cluster Associated with *Uromyces appendiculatus* Infection in Common Bean *Phaseolus vulgaris* L.

**DOI:** 10.3390/ijms18061109

**Published:** 2017-05-23

**Authors:** Antonette R. Todd, Nicole Donofrio, Venkateswara R. Sripathi, Phillip E. McClean, Rian K. Lee, Marcial Pastor-Corrales, Venu (Kal) Kalavacharla

**Affiliations:** 1 Department of Agriculture and Natural Resources, Delaware State University, Dover, DE 19901, USA; atodd@desu.edu (A.R.T.); v.sripathi@aamu.edu (V.R.S.); 2 Department of Plant and Soil Sciences, University of Delaware, Newark, DE 19716, USA; ndonof@udel.edu; 3 Center for Molecular Biology, Department of Biological & Environmental Sciences, College of Agricultural, Life & Natural Sciences, Alabama A&M University, Normal, AL 35762, USA; 4 Department of Plant Sciences, North Dakota State University, Fargo, ND 58105, USA; phillip.mcclean@ndsu.edu (P.E.M.); rian.lee@ndsu.edu (R.K.L.); 5 United States Department of Agriculture-Agricultural Research Service, Soybean Genomics and Improvement Laboratory, Beltsville Agricultural Research Center, Beltsville, MD 20170, USA; talo.pastor-corrales@ars.usda.gov; 6 Center for Integrated and Environmental Research (CIBER), Delaware State University, Dover, DE 19901, USA

**Keywords:** common bean, bean rust, phaseolus, uromyces, RNA-seq, transcriptome, marker assisted profiling, delineation

## Abstract

Common bean (*Phaseolus vulgaris* L.) is an important legume, useful for its high protein and dietary fiber. The fungal pathogen *Uromyces appendiculatus* (Pers.) Unger can cause major loss in susceptible varieties of the common bean. The *Ur-3* locus provides race specific resistance to virulent strains or races of the bean rust pathogen along with *Crg*, (Complements resistance gene), which is required for *Ur-3*-mediated rust resistance. In this study, we inoculated two common bean genotypes (resistant “Sierra” and susceptible crg) with rust race 53 of *U. appendiculatus*, isolated leaf RNA at specific time points, and sequenced their transcriptomes. First, molecular markers were used to locate and identify a 250 kb deletion on chromosome 10 in mutant crg (which carries a deletion at the *Crg* locus). Next, we identified differential expression of several disease resistance genes between Mock Inoculated (MI) and Inoculated (I) samples of “Sierra” leaf RNA within the 250 kb delineated region. Both marker assisted molecular profiling and RNA-seq were used to identify possible transcriptomic locations of interest regarding the resistance in the common bean to race 53. Identification of differential expression among samples in disease resistance clusters in the bean genome may elucidate significant genes underlying rust resistance. Along with preserving favorable traits in the crop, the current research may also aid in global sustainability of food stocks necessary for many populations.

## 1. Introduction

The common bean is essential for global food sustainability, as it is nutritionally and economically important in many developing and developed countries throughout the world, such as eastern Africa [1] and Latin America [2]. Worldwide, more than 12 million metric tons of beans are produced annually, with most (5.5 million metric tons) being produced in Latin America [2]. The common bean’s crop value in the US alone is more than 1 billion dollars [3]. Along with its economic importance, common bean is also generally high in protein, providing about 30% of the recommended daily protein intake [4], which accounts for approximately 22% of its weight. The fiber content aids in stabilizing blood sugar and cholesterol, assisting in the fight against obesity and diabetes [5]. Not only does common bean supply nutrients like proteins, carbohydrates, and vitamins [6], it can also be stored for an extended period of time in its dry form, contributing to cost effectiveness. Diverging from a common ancestor over 100,000 years ago [7], two distinct groups of common beans, known as the Mesoamerican and Andean gene pools, were independently domesticated more than 8000 years ago in Central and South America [8].

The fungal pathogen *Uromyces appendiculatus* (Pers.) Unger causes the rust disease that negatively affects common bean production. The symptoms of rust disease appear as pustules that develop on susceptible varieties. As an obligate biotroph, *U. appendiculatus* needs a living, susceptible plant host in order to propagate and it cannot be cultured. *Uromyces appendiculatus* flourishes in temperatures between 17–25 °C with high humidity (>95%). In optimal conditions, the fungal pathogen can cause major crop losses.

Common bean cv “Sierra” was the first of many pinto bean varieties released from Michigan State University and USDA-ARS [9]. It was first released in the early 1990s after testing for eight seasons in over forty locations. “Sierra” was bred using several varieties and breeding lines, including pinto, navy and black beans. Beans were selected for traits such as seed size, color, growth habit, and resistance to races of rust that are common in Michigan [9]. Mutant crg was one of three mutants derived from fast neutron bombardment of “Sierra” seeds. The goal was to identify rust resistance genes by genetically comparing rust resistant plants to rust susceptible plants. Unlike its progenitor, crg is susceptible to race 53 rust [10].

In order to combat pathogenicity, plants, such as common bean, have adapted two major types of defense: pathogen associated molecular pattern (PAMP) triggered immunity (PTI) and effector triggered immunity (ETI) [11]. Both defenses play important roles in protecting plants from pathogen attacks and providing resistance. PTI triggers innate immunity, and is associated with low resistance. PAMPs are crucial components specific to pathogens, therefore they allow the host to distinguish itself from the pathogen and activate the innate immunity response [12]. PAMPs are also widely conserved in pathogens and are necessary for their survival [13]. Pathogens will deploy effector proteins into the host, eliciting ETI. ETI is more durable than PTI and is recognized by resistance (R) proteins in the host, as described in the zig zag model [11].

During PTI, proteins such as flagellin, a prokaryotic elongation factor (EF-Tu), and chitin are recognized by receptor-like kinases (RLK) [14]. These proteins are recognized by immune receptor complexes that include EF-Tu Receptor (EFR), which recognizes EF-Tu, Chitin Elicitor Receptor Kinase 1 (CERK1), recognizes chitin, and Flagellin-sensitive 2 (FLS2), which recognizes flagellin [14]. Once PTI is overcome, ETI is activated by proteins in the host that monitor for modifications caused by the pathogen. Nucleotide binding oligomerization domain (NOD)-like receptors (NLRs) are responsible for guarding host proteins that attract effectors [15].

The *Ur-3* locus in common bean confers resistance to race 53, as well as over 40 other races of *U. appendiculatus*. This locus was first identified and described by Ballantyne in the late 1970s [16]. *Ur-3* is present in Mesoamerican landraces of common bean including Aurora and Mex235. It has been genetically linked to chromosome 11 in common bean [17]. Along with the *Ur-3* locus, the *Crg* locus is required for resistance to race 53 [10]. The *Crg* locus was first described by Kalavacharla et al. [10] in the study of irradiated common bean seed progeny. *C*omplements *r*esistance *g*ene (*Crg*), was first identified in one of three mutants with susceptibility to race 53 rust.

In this study, we seek to better understand the interaction between the *Ur-3* resistance locus in common bean and race 53 by identifying the location of the *Crg* gene. We employ methods that help identify and characterize disease resistance gene clusters in common bean, including marker-assisted molecular genetics, deletion mutant analysis, and high-throughput sequencing (RNA-seq). Additionally, we used bioinformatics to identify differentially expressed genes within chromosome regions containing disease resistance signatures such as Nucleotide Binding Sites (NBSs) and Leucine Rich Repeats (LRRs). We believe identification of differentially expressed genes among resistant and susceptible genotypes of bean may assist in identifying rust resistance genes in common bean, particularly at the *Crg* and *Ur-3* loci.

## 2. Results

### 2.1. Marker Assisted Molecular Profiling of Genomic DNA

**Figure 1:**
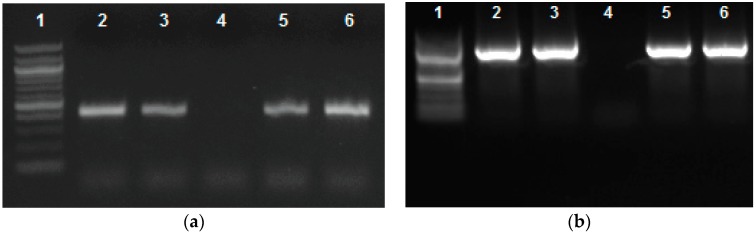
SB1 amplification pattern. (**a**) PCR using primers for molecular markers for SB1. 1. 100 bp ladder; 2. “Sierra”; 3. “Olathe”; 4. crg; 5. ur3-∆2; 6. ur3-∆3; (**b**) PCR using primers for molecular markers for *010G025000* 1.100 bp ladder; 2. “Sierra”; 3. “Olathe”; 4. crg; 5. ur3-∆2; 6. ur3-∆3. The amplifications are missing in crg but are present in all other genotypes, indicating that a mutation associated with susceptibility to race 53 is in this region.

**Figure 2:**
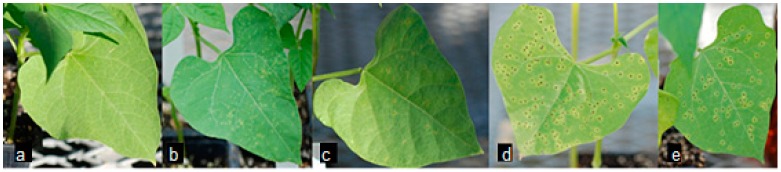
Five genotypes of common bean inoculated with race 53 of the fungal rust pathogen Uromyces appendiculatus. (**a**) Race 53 resistant “Sierra”; (**b**) Race 53 susceptible “Olathe”; (**c**) Race 53 susceptible mutant crg; (**d**) Race 53 susceptible ur3-∆2; (**e**) Race 53 susceptible ur3-∆3. “Sierra” shows no sign of pathogen growth. All susceptible genotypes vary in degree of uredia (rust pustules) formation, with mutant crg exhibiting the mildest reaction.

**Figure 3:**
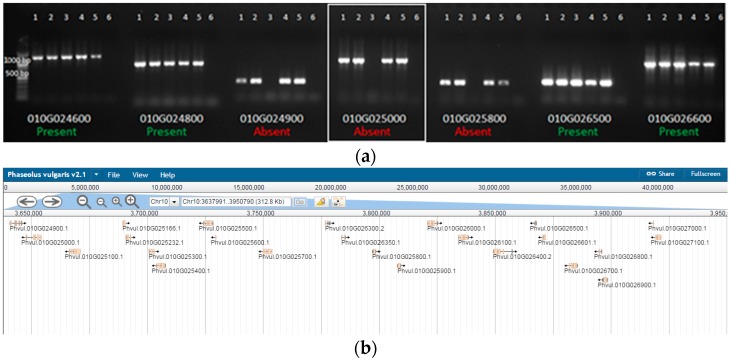
Delineated region on chromosome 10. (**a**) Two hundred and fifty kb delineated deletion region on chromosome 10. PCR using seven different primer sets from the region spanning the deletion region in mutant crg. 100 bp ladder; 1. “Sierra” 2. “Olathe” 3. crg 4. ur3-∆2 5. ur3-∆3 6. water control. The boxed region contains the resistance gene analog SB1; (**b**) Alignment of transcripts within the delineated region as presented on the Phytozome website.

The use of molecular markers allows for the differentiation of genomic DNA amplification among genotypes. Previous research demonstrated that the resistance gene analog (RGA) marker SB1 amplifies in “Sierra” genomic DNA [18], but not crg genomic DNA [10], as seen in Figure 1a. The location of this marker was recently identified by using the Basic Local Alignment Search Tool (BLAST) on the common bean genome. An earlier version of a common bean global transcriptome was generated using 454 sequencing on non-inoculated “Sierra” [19]. More recently, a common bean genome and transcriptome, which are publicly available at Phytozome [20], were generated using the Andean landrace G19833 or “Chaucha Chuga” [8]. The SB1 marker aligns to a single locus on chromosome 10, and is associated with a single gene (*Phvul.010G025000*) in common bean. Primers were designed from gene *Phvul.010G025000* to confirm amplification in “Sierra” (Figure 1b). We used five common bean genotypes, including three “Sierra”-derived mutants in this study (Figure 2). Primers were designed outwardly, specifically from genes adjacent to *Phvul.010G025000* as opposed to intronic areas and regions farther away. First, genomic DNA was amplified using primers designed from a gene directly beside the SB1-containing *Phvul.010G025000*, *Phvul.010G024900*. Subsequent primer sets were designed from genes adjacent to the location of *Phvul.010G025000* (Table 1) to determine the size of the missing region in crg. We obtained primer sets with the same amplification/deletion pattern as *Phvul.010G025000* (Figure 3). “Sierra” and crg differ only on chromosome 10 in a region spanning less than 250 kb. RNA-seq data were analyzed in the region of the deletion on chromosome 10.

### 2.2. Illumina Sequence Data

RNA-seq was used to obtain transcriptomes for five inoculated (I) and mock-inoculated (MI) genotypes of bean (Figure 1). Two genotypes, race 53 resistant cultivar “Sierra” (which contains the *Ur-3* gene), and the “Sierra”-derived susceptible mutant, crg, were the focal points of this study. RNA-seq data were generated from a rust race 53 inoculation time course study in common bean. We analyzed transcriptomes for two biological replicates of “Sierra” and crg, including 12 h post inoculation (HPI) MI and I samples for each of the two replicates, totaling eight libraries of 175 million reads (Table 2). In common bean [10], soybean (*Glycine max*) [19], and related studies [21], hypersensitive response (HR) has been shown to be expressive between 0–12 hpi. Therefore, 12 hpi is the time-point chosen for our study [22]. Reads were mapped to the publicly available common bean transcript genome version 1 [20] and were analyzed using CLC Genomics Workbench (Qiagen, København, Denmark). Mapped read samples of “Sierra” and crg were compared to one another to determine differential gene expression across the entire transcriptome. We were interested in finding significant expression variance between “Sierra” MI and “Sierra” I at the probable *Crg* locus. Therefore, we employed a marker assisted molecular profiling method using genomic DNA from “Sierra” and crg to identify differential amplification patterns. Marrying the two approaches allowed the deduction of the deletion region in crg, which permitted a focal point on a candidate gene area for *Crg*.

### 2.3. Gene Expression during U. appendiculatus Infection

To characterize differential gene expression at the candidate Crg locus, deduced by genomic DNA PCR, we analyzed the RNA-seq data of “Sierra” MI, “Sierra” I, and crg I against each other in the form of a heat map (Table 3). Table 3 indicates that there is no expression of this cluster of genes in mutant crg. Among “Sierra” MI and “Sierra” I, there is differential expression in several genes, most notably, *Phvul.010G025800*, which is currently a gene of unknown function.

The group of genes present in the delineated region of chromosome 10 mostly belongs to the disease resistance protein Toll/Interleukin- Nucleotide Binding Site- Leucine Rich Repeat (TIR-NBS-LRR class) family. This cluster includes a mitochondrial processing peptidase beta subunit insulinase protein (MPPBETA) belonging to the Peptidase family M16, an NB-ARC (Apaf-1, R-proteins, and CED-4) [23] domain-containing disease resistance protein, target of AvrB operation1 (TAO1), and three proteins of unknown function (Table 1). TAO1 is a disease resistance protein induced by the AvrB effector in *Pseudomonas syringae* [24]. The TAO1 protein works in tandem with RPM1 plants, a gene conferring resistance to *P. syringae* in Arabidopsis and soybean [24]. It also works with Pto in RPM1 plants, which also confers resistance to *P. syringae* in tomato and was the first R gene cloned that followed Flor’s gene-for-gene theory [25]. It is required for full resistance against the DC3000 (avrB) strain of *P. syringae* [24].

The NB-ARC protein domain regulates *R* gene activity [26]. It also serves as a signaling motif in plant cell regulatory systems [23]. It is instrumental in programmed cell death, which may be one of the main factors in hypersensitive resistance (HR) response in the race 53 rust resistant plant, “Sierra”. The NB-ARC domain is also present in at least five other plant Resistance *R* genes, including RPM1 [27], RPS2 [28,29] RPP5 [30], N [31], and L6 [32]. All of the mentioned genes are also encoded with a C-terminal end of leucine rich repeats (LRR). They are then divided into two groups, differing only at the N-terminal, which are composed of either leucine zippers (RPS2 and RPM1) or Toll/Interleukin-1 (N, L6, and RPP5).

### 2.4. RT-PCR and Confirmation of Gene Expression

**Figure 4:**
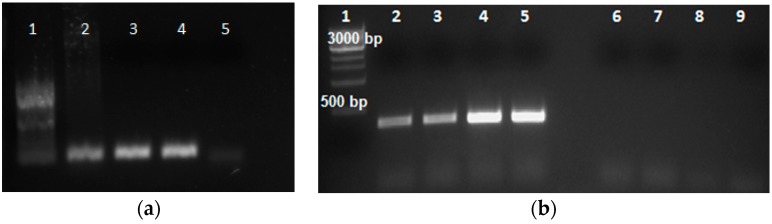
(**a**) PCR showing constitutive amplification of endogenous gene *Cons 7*. 1. 100 bp ladder; 2. Genomic “Sierra” DNA; 3. “Sierra” MI; 4. “Sierra” I; 5. water control; (**b**) cDNA PCR with select primer set designed from *Phvul.010G025800* reveals increased expression in “Sierra” I versus “Sierra” MI. There is no amplification in any of the four mutant crg samples. 1. 1 kb ladder; 2. “Sierra” MI; 3. “Sierra” MI; 4. “Sierra” I; 5. “Sierra” I; 6. crg MI; 7. crg MI; 8. crg I; 9. crg I.

Reverse transcriptase PCR (RT-PCR) was conducted using identical tissue for RNA-seq to generate cDNAs from “Sierra” MI, “Sierra” I, crg MI, and crg I to test primers within the delineated region on chromosome 10. Primers designed from gene *Phvul.010G025800* were used to perform PCR in order to visualize differences between all cDNA samples (Figure 4). No amplification was apparent in any crg samples, MI or I. However, there was a difference in amplification intensity between the two “Sierra” samples, MI and I. The inoculated “Sierra” sample amplified brighter band intensity than the mock inoculated “Sierra” sample. Therefore, we confirmed that inoculated leaf tissue expresses this particular gene more strongly than mock inoculated tissue.

### 2.5. Real Time/Quantitative PCR (q-PCR)

**Figure 5:**
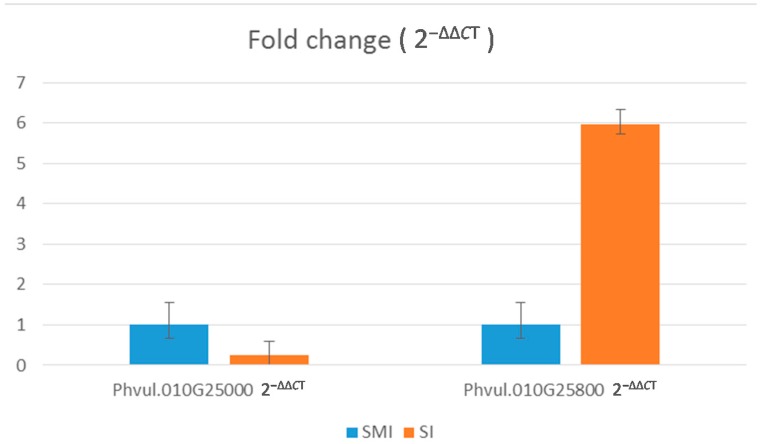
Fold change comparison of q-PCR data from genes *Phvul.010G025000* and *Phvul.010G025800*. The data presented in this figure exhibits the differential expression between “Sierra” MI and I samples for both genes.

To further confirm differential expression between “Sierra” MI and “Sierra” I, q-PCR (Real Time/Quantitative PCR) was performed using primers designed from genes in the delineated region (Table S1). Among all primer sets used from within the delineated region, the most highly expressed gene was *Phvul.010G025800* (Table 3), which corresponds with expression data in Table 4. q-PCR data were scored for expression using cycle threshold (*C*_T_) values. In quantitative PCR, *C*_T_ is defined as the number of PCR cycles needed for the fluorescent signal to cross the threshold. When there is more target nucleic acid present, the fluorescent signal crosses the signal sooner, resulting in a lower cycle number. Lower *C*_T_ values correlate with a higher abundance of target nucleic acid. The high *C*_T_ value in samples amplifying target *Phvul.010G025000* correlate with low to no expression. This comparison is shown in fold change values generated from q-PCR (Figure 5).

## 3. Discussion

### 3.1. Identification of SB1 Location in Gene Phvul.010G025000

The release of the common bean genome has allowed information to be acquired that was previously unavailable. Hence, we were able to compare the SB1 molecular marker sequence to identify its location in the genome. Molecular marker SB1 amplifies in all genomic DNA samples except for crg. For this reason, the focal point included the regions to the left and right of the SB1 containing *Phvul.010G025000* to identify the genes involved in crg’s susceptibility to rust race 53 of the bean rust pathogen. When using MI and I cDNA from “Sierra” and crg, we found that *Phvul.010G025000* does not amplify in either group of samples. This is in agreement with information provided by Phytozome [20]; this gene only amplifies in the roots and nodules of common bean genotype G19833. Therefore, we conclude that *Phvul.010G025000* is most likely not the *Crg* gene, although it is probably located in the vicinity. Alternately, *Phvul.010G025000* may not express at the selected 12 hpi time point. In order to positively confirm this, additional time course studies are needed in the future. This includes collecting samples at closer time points after initial inoculation (ex. 0–2 hpi, 2–4 hpi, etc.).

### 3.2. Delineation of crg Deletion Region

We were able to identify clusters of differentially expressed genes among genotypes of mock inoculated versus inoculated samples. The most encouraging find occurred in the disease resistance gene cluster on chromosome 10, which is missing in rust race 53 susceptible crg. Through genomic DNA PCR and reverse transcriptase PCR, we were not only able to delineate the 250 KB region on chromosome 10, but were also able to detect expression in “Sierra” and no expression in crg, while delineating the region of interest in combination with molecular marker SB1. Differential expression of a cluster of disease resistance genes was shown between “Sierra” MI and “Sierra” I.

The unknown gene within the delineated region, *Phvul.010G025800*, was most differentially expressed between “Sierra” MI and I. However, it does not contain a disease resistance-related conserved domain, such as NBS, TIR, LRR, and its role is not yet understood. According to the conserved domain search on the National Center for Biotechnology Information (NCBI) website [33], part of the sequence is a probable serine/threonine-protein kinase. Serine/threonine kinases are known to be active in signal transduction [25]. If *Phvul.010G025800* truly contains a serine/threonine kinase domain, this may explain the gene’s role in signal transduction between fungal pathogen effectors and host genes. Also within this region is the TAO1 gene, which serves as a target of operation of the avirulence gene B in *P. syringae*. Amplification of cDNA paired with RT-PCR confirms that TAO1 does not express in either “Sierra” sample (MI or I) presented in this study. Similarly, the unknown gene *Phvul.010G025800* may be a target for effectors from avirulence genes in *U. appendiculatus* in order to contribute to rust resistance. Furthermore, since this study primarily included data from 12 hpi only, the expression levels for TAO1 or *Phvul.010G025800* are not completely known throughout the initiation of the bean-bean rust infection.

Although the deletion region was delineated in crg by using genomic PCR, reverse transcriptase PCR, and RNA-seq, our main focus in this study was to identify differential expression between “Sierra” MI and “Sierra” I samples when challenged with rust. Since the aim was not to perform a global study on “Sierra” MI and I, the mutated genotypes were used to identify fast-neutron derived break-points in the genome of susceptible plants. In this way, the focus is on a smaller subset of genes, giving a greater chance at identifying the gene(s) of interest. Three key points were concluded through this research. One, SB1 is located on chromosome 10, not chromosome eight as previously believed. Two, SB1 is part of a gene, but does not express in leaf tissue at our specific time point (12 hpi). Three, the deletion in crg is less than 300 kb, making the list of identified genes missing less than twenty.

### 3.3. Current Perspective of Disease Resistance and Relation to the Bean-Bean-Rust Interaction

Plant-pathogen interactions have been a necessary study to understand the relationships and mechanisms that drive disease resistance [34]. For more than a hundred years, people have used methods such as breeding to try to control immune receptors in plants [35]. The number of cloned *R* genes has greatly increased over the last 20 years. As more plant *R* genes are isolated and cloned, there is new promise that scientists may obtain a better understanding of pathogenicity and gene function, which can ultimately be used to create more resistant plant varieties. The identification and isolation of *R* genes is advantageous, as developing disease resistant varieties in plants may be a more suitable approach than the use of pesticides to manage virulent pathogens on crops. Since the long-term effects of pesticide use are not completely known, many consumers now opt for organically raised crops. While humans have been purposely studying *R* genes for a hundred years, pathogens have been evolving along with their hosts for much longer [35].

Disease resistance genes usually contain signature motifs that are recognizable as such. It is understood that NBS, LRR, and TIR domains are normally present in *R* genes, making them easy to identify in a group of amino acid sequences. However, recent work by two groups, [36,37], reveals unusual domains anchoring themselves to nucleotide binding oligomerization domain (NOD)-like receptors (NLRs) [15]. One of these unusual domains is the WRKY-like or WRKY transcription factor-like domain that is fused to the *RPS1* gene in *Arabidopsis*. The RPS1 and RPS4 complex is paired with the WRKY domain to provide recognition of two bacterial effectors. However, when the WRKY-like domain anchors itself to this complex instead, the bacterial effectors never interact with the true target. Therefore, instead of pathogen effectors being intercepted by true WRKY domains, they are intercepted by an imposter. Hence the WRKY-like domain attracts the pathogen effectors and suppresses the immune response [36,37]. Current plant disease perspectives seek to use the pathogen effectors as a means to exploit *R* gene mediated resistance [38]. Initially, pathogen effectors were believed to directly interact with host *R* genes. It is now understood that the primary role of pathogen effectors is to alter the host’s cellular function and to create a better environment for itself [39]. The hemibiotrophic oomycete pathogen *Phytophthora infestans* secretes the effector protein AVR3a into the host. There are two forms of AVR3a, which are AVR3aKI and AVR3aEM. AVR3aKI strongly suppresses infestin 1 (INF1) induced cell death (ICD) in the potato interacting with the R3a protein. AVR3aEM weakly suppresses INF1 ICD when interacting with R3a [40]. This interaction suppresses HR by recognition of the R3a protein in the host by AVR3a [41].

In obligate biotrophs, such as *Puccinia graminis* and *Melampsora larici*, two advances in genome sequencing have allowed the identification of predicted gene sequences, which also include conserved domains present in powdery mildew [42]. This conserved domain consists of an 8-cysteine or 10-cysteine pattern. Observing conserved domains among pathogens may be the key to identifying effector function.

Bean-bean rust interaction research continues to be an important topic as pathogens evolve along with hosts *R* genes. Most recently, the rust resistant *Ur-3* gene has been fine mapped to an approximately 47 kb region on the lower arm of chromosome 11 in common bean [43]. Along with this finding, several molecular markers have been developed, including the SS68 KASP (Kompetitive Allele Specific PCR) marker that is believed to be tightly linked to the *Ur-3* gene. The development of new molecular markers will help in screening potential new cultivars for rust resistance genes.

The research presented in this study explains the necessity of continually identifying disease resistance genes in food crops as a means of obtaining global food sustainability. As pathogens eventually overcome known disease resistance gene, it is important to isolate new genes that confer resistance for the sake of the global population’s nutrition and economy. As the population continues to grow, it is necessary to make sure we can continue to produce food, especially in places where particular crops are relied on heavily.

## 4. Materials and Methods

In this study, five common bean genotypes were used, which includes rust race 53 resistant “Sierra”, rust race 53 susceptible “Olathe”, and three rust race 53 susceptible mutants crg, ur3-Δ2 and ur3-Δ3, which were derived from “Sierra”. Additionally, for verification of IDT designed primers (Integrated DNA Technology, Coralville, IA, USA), common bean genotype G19833 was used for in-silico PCR [44]. Plants were grown in a greenhouse at Delaware State University (Dover, DE, USA) as per standard conditions to collect leaves for isolating DNA. Plants were grown for 10 days to allow for the emergence of primary leaves. After collection, plants were maintained for seed growth and propagation.

### 4.1. DNA Isolation

Leaves were collected by genotype and flash frozen in liquid nitrogen and stored at −80 °C until use. DNA isolation was conducted using a CTAB method [45], followed by DNA phenol: chloroform clean up [46].

### 4.2. DNA Quantification and Marker Analysis

After extraction, all DNAs were quantified by NanoDrop 2000 spectrophotometer (Thermo Scientific, Waltham, MA, USA), and diluted to a standard concentration of 100 ng/μL. Simultaneously, the DNAs were quantified by gel electrophoresis to ensure that the quantity and quality of each genotype can be visualized in agarose. We performed PCR with molecular markers SB1 and SK14 (Figure S1) to differentiate the five genotypes (Table S2). Each 25 μL PCR reaction contained 16.25 μL of distilled autoclaved water, 5 μL of 5× taq buffer with MgCl_2_, 1 μL each of forward and reverse primers, 0.50 μL of dNTPs, 0.25 μL of taq polymerase, and 1 μL of DNA. The optimized PCR protocol for SK14 amplification was 34 cycles of 10 s at 94 °C, 40 s at 63 °C, 2 min at 72 °C, 1 cycle of 5 min at 72 °C, and a final holding cycle at 4 °C. The PCR protocol for molecular marker SB1 was 1 cycle of 94 °C for 3 min, 30 cycles of 94 °C for 1 min, 60 °C for 1 min, and 72 °C for 2 min, 1 cycle of 72 °C for 5 min. PCR amplifications were visualized on a 1% agarose gel containing 0.008% ethidium bromide staining solution.

Once all genotypes were confirmed via PCR, seed collected from each of the original plants were planted and labeled accordingly. Plants were grown in the greenhouse during early spring under seasonal conditions.

### 4.3. Maintenance of Pathogen and Inoculum Preparation

Original urediospores received from USDA-ARS (Beltsville, MD, USA) were stored at −80 °C. A 0.1% Tween 20 solution was prepared to serve as a surfactant and spreader for the fungal spores. The amount of Tween 20 solution was divided to accommodate both inoculating and mock inoculating solutions. Urediospores were added to one aliquot of Tween 20 solution and spun for at least 3 h before inoculations. The final volume of urediospores in the inoculating solution was quantified to 20,000 spores per milliliters. This amount was quantified by using a hemocytometer and light microscope. A total of 50 mL of urediospore solution was used for 15 plants.

### 4.4. Plant Inoculation

The seeds were germinated in Petri dishes (Thermo Scientific) prior to being planted in soil pots. Germination usually took two to three days. Once plants were transferred to pots, they were first grown in the greenhouse. Prior to being spray inoculated, plants were placed in a Conviron Growth room under 12 h photoperiod (daylight) conditions. Plants were rotated within flats to guarantee that each plant received an equal distribution of light and temperature fluctuation. Once plants were ready for inoculation, approximately 10 days after germination began, the growth room humidity was adjusted to 95–100% and the lights were turned off. Plants were sprayed with inoculum on both leaf surfaces evenly. Inoculated plants were placed in the growth room’s humidity chamber for 16 h overnight in the dark.

A total of two biological replicates were inoculated on two separate days in this study. Garden five plants were inoculated with rust race 53 approximately 10 days after germination, consisting of the inoculation of the first two leaves that emerged after the cotyledon. Plants were rested for five minutes to allow inoculum to dry. Zero hpi samples were collected from both inoculated (I) and mock inoculated (MI) plants and were flash frozen in liquid nitrogen before being stored in the −80 °C freezer. Plants were placed in a humidity chamber with >95% humidity at 19 °C. Samples were collected again at 12 hpi and 84 hpi, flash frozen, and kept at −80 °C. Before further processing, plants were evaluated for uredia growth. Uredia growth was monitored until urediospore content caused them to burst. Urediospores were collected by placing a clean sheet of aluminum foil beneath the infected leaves. Infected leaves were lightly tapped to release the urediospores. Collected urediospores were validated using a light microscope and stored in a 1.5 mL micro centrifuge tube in the −80 °C freezer.

### 4.5. RNA Isolation

Samples that were collected at 12 hpi were processed further for RNA isolation. Isolations were performed using the TRIzol method (Invitrogen, Carlsbad, CA, USA). 1 mL of TRIzol Reagent was added per 50–100 mg of tissue sample. The tissue was ground in a micro centrifuge tube with micro pestles. The samples were kept at room temperature for five minutes in a micro centrifuge tube. Two hundred microliters of chloroform were added to each tube and mixed well. They were incubated again for another two to three minutes. The samples were then centrifuged at 12,000 rpm in an ultra-centrifuge for 10 min at 4 °C. After centrifugation, the top layer was pipetted into a separate tube for further cleaning. Five hundred microliters of 100% isopropanol were added to each tube and incubated at room temperature for 10 min. Next, each tube was centrifuged for 10 min at 4 °C. After removing the supernatant from each tube, each pellet was washed with 1 mL of 75% ethanol. The samples were briefly vortexed, and then centrifuged again to remove any excess traces of TRIzol or chloroform. The supernatant was carefully removed by pipetting and the pellet was dried almost completely. RNA was quantified by gel electrophoresis, NanoDrop 2000 (Thermo Scientific), and Qubit Fluorometer (Invitrogen).

### 4.6. cDNA Synthesis

For quality control purposes, RNA was then converted to cDNA using ProtoScript II (NEB, Ipswich, MA, USA). Approximately 1 µg of RNA was mixed with d (T) 23 VN (50 µM) nucleotides and nuclease free water and incubated at 65 °C for five minutes. The tube was immediately placed on ice to keep the RNA denatured. 10 µL of ProtoScript II Reaction Mix (2×) and 2 µL of ProtoScript II Enzyme Mix (10×) was added to the denatured RNA for a total of 20 µL and incubated at 42 °C for one hour. Samples were incubated at 80 °C to inactivate the enzyme. Synthesized cDNA was then quantified by NanoDrop and analyzed through PCR. PCR analysis with molecular marker SK14 primers and SB1 primers was carried out to confirm that the cDNA was free of genomic DNA contamination. We also used a set of primers derived from cDNA PCR with soybean (*Glycine max*). Constitutive gene 7, or cons 7, is one of several genes in soybean that always expresses. The cons 7 gene was used to check the integrity of RNA isolation.

### 4.7. Illumina Library Prep

Total RNA was isolated to build sequencing libraries with TruSeq RNA Sample Preparation Kit v2 (Illumina, San Diego, CA, USA). Library prep included the purification and fragmentation of mRNA from total RNA, first and second strand cDNA synthesis, end repair, adapter ligation, PCR amplification, library validation, normalization and pooling. The experiment included two treatments, two replicates and two genotypes, resulting in eight libraries. The libraries are labeled “Sierra” MI Rep 1, “Sierra” MI Rep 2, “Sierra” I Rep 1, “Sierra” I Rep 2, crg MI Rep 1, crg MI Rep 2, crg I Rep 1, and crg I Rep 2. Samples were pooled and sequenced on Illumina HiSeq 2500 platform generating single-end 50 nucleotide reads. Libraries were sequenced at the University of Delaware Sequencing & Genotyping Center at Delaware Biotechnology Institute (Newark, DE, USA). Sequences were submitted to the Sequence Read Archive (SRA) at NCBI under BioProject number PRJNA369418.

### 4.8. Data Analysis

Sequencing reads were analyzed using CLC Genomics workbench v7 (Qiagen, København, Denmark). Each set of reads were processed individually using the RNA-seq mapping tool. Reads were mapped to the common bean transcriptome and genome v1. Expression values were calculated using Reads per Kilobase of transcript per Million mapped reads (RPKM). “Sierra” and crg (MI and I) samples were compared to identify differential expression among genes specifically on chromosome 10 at the location of molecular marker SB1. Subsequently, “Sierra” MI and I were compared to identify differential expression in the delineated region. Heat maps were generated to visually compare regions of interest on chromosome 10 in “Sierra” and crg. Additional primer sets for genes surrounding the molecular marker SB1 were designed using the Integrated DNA Technology Primer Quest tool (IDT, Coralville, IA, USA).

### 4.9. Real Time/Quantitative PCR (q-PCR)

For q-PCR, we used the Applied Biosystems 7500 Real Time platform and Power SYBR green Master mixes. cDNAs were quantified to 100 ng/µL in each sample before analysis. Twenty-five µL reactions prepared for each processed sample including 12.5 µL of Power SYBR Master mix, 1 µL each of forward and reverse primer, 1 µL of cDNA and 9.5 μL of nuclease free water. *Cons 7* was used as an endogenous internal control for each q-PCR run. There were three technical replicates quantified for each sample.

## Figures and Tables

**Table 1:** List of candidate genes in delineated region with corresponding gene function.

Gene Name	Function
*Phvul.010G024900*	MPPBETA Insulinase (Peptidase family M16)
*Phvul.010G025000*	Disease resistance protein (TIR-NBS-LRR class)
*Phvul.010G025100*	NB-ARC domain-containing disease resistance
*Phvul.010G025200*	Disease resistance protein (TIR-NBS-LRR class)
*Phvul.010G025300*	Disease resistance protein (TIR-NBS-LRR class)
*Phvul.010G025400*	Disease resistance protein (TIR-NBS-LRR class)
*Phvul.010G025500*	Disease resistance protein (TIR-NBS-LRR class)
*Phvul.010G025600*	Unknown
*Phvul.010G025700*	Disease resistance protein (TIR-NBS-LRR class)
*Phvul.010G025800*	Protein of unknown function (DUF506)
*Phvul.010G025900*	Mitochondrial transcription termination factor
*Phvul.010G026000*	Disease resistance protein (TIR-NBS-LRR class)
*Phvul.010G026100*	Disease resistance protein (TIR-NBS-LRR class)
*Phvul.010G026200*	Disease resistance protein (TIR-NBS-LRR class)
*Phvul.010G026300*	target of AVRB operation1
*Phvul.010G026400*	Disease resistance protein (TIR-NBS-LRR class)
*Phvul.010G026500*	Unknown

**Table 2:** RNA-seq data for total reads sequenced and mapped to the common bean transcriptome for “Sierra” and crg samples, both mock-inoculated (MI) and inoculated (I).

Sample	Replicate	Total Reads	Total Reads Mapped	% Reads Mapped
“Sierra” MI	Replicate 1	29,470,433	26,360,691	89.44
“Sierra” MI	Replicate 2	15,458,316	13,419,654	86.81
“Sierra” I	Replicate 1	21,564,405	18,746,890	86.93
“Sierra” I	Replicate 2	20,849,023	18,915,928	90.73
crg MI	Replicate 1	10,843,510	8,978,807	82.80
crg MI	Replicate 2	19,146,153	16,169,339	84.45
crg I	Replicate 1	30,918,507	27,509,416	88.97
crg I	Replicate 2	27,518,359	24,014,203	87.27

**Table 3:** Heat map comparison of crg MI vs. crg I vs. “Sierra” MI vs. “Sierra” I. Genes are listed by reads per kilobase of transcript per million mapped reads (RPKM) expression value and are in the delineated region. The green values indicate little to no expression. Yellow values indicate moderate expression. Red indicates high expression. *Phvul.010G025800* is most highly expressed and most differentially expressed across samples.

Gene Name	crg MI	crg I	“Sierra” I	“Sierra” MI
*Phvul.010G024900*	0	0	0.45	1.29
*Phvul.010G025000*	0	0	0	0.01
*Phvul.010G025100*	0	0	0.01	0.01
*Phvul.010G025200*	0	0	0.11	0.16
*Phvul.010G025300*	0	0	0.13	0.14
*Phvul.010G025400*	0.038922	0.05	5.28	4.91
*Phvul.010G025500*	0.039883	0.01	1.76	2.71
*Phvul.010G025600*	0.039755	0	0.44	0.94
*Phvul.010G025700*	0.487667	0	0	0
*Phvul.010G025800*	0	0.03	18.7	13.3
*Phvul.010G025900*	0.162343	0	0.62	1.62
*Phvul.010G026000*	0	0	1.9	1.79
*Phvul.010G026100*	0	0.02	3.16	3.43
*Phvul.010G026200*	0	0	3.12	2.87
*Phvul.010G026300*	0	0.06	0.36	0.1
*Phvul.010G026400*	0	0.02	2.88	2.92
*Phvul.010G026500*	0	0	0.08	0

**Table 4:** q-PCR with “Sierra” MI and “Sierra” I samples and primer sets designed from delineated region. Lower cycle threshold (*C*_T_) values indicate higher abundance of target nucleic acid, i.e., higher expression. 3a.Endogenous gene *Cons 7* and *Phvul.010G025000*. *Cons 7* is constitutively expressed in MI and I samples. High *C*_T_ values in MI and I *Phvul.010G025000* samples indicate low expression 3b. The lowest *C*_T_ values in the table are associated with gene *Phvul.010G025800*, particularly in “Sierra” I samples.

Sample Name	Target Name	Reporter	Cycle Threshold	*C*_T_ Mean	ΔΔ*C*_T_	2^−ΔΔ*C*T^
**3a**						
“Sierra” MI	*Cons 7*	SYBR	28.87731361			
“Sierra” MI	*Cons 7*	SYBR	28.26262856	28.64316559		
“Sierra” MI	*Cons 7*	SYBR	28.78955269			
“Sierra” I	*Cons 7*	SYBR	28.02788353			
“Sierra“ I	*Cons 7*	SYBR	28.2238121	28.2289753		
“Sierra” I	*Cons 7*	SYBR	28.43523407			
“Sierra” MI	*Phvul010G025000*	SYBR	34.58884		0	1
“Sierra” MI	*Phvul010G025000*	SYBR	34.19945	34.89832	0	1
“Sierra” MI	*Phvul010G025000*	SYBR	35.90666		0	1
“Sierra” I	*Phvul010G025000*	SYBR	35.85514		2.053371	0.24092
“Sierra” I	*Phvul010G025000*	SYBR	36.9123	36.5375	2.053371	0.24092
“Sierra” I	*Phvul010G025000*	SYBR	36.84506		2.053371	0.24092
**3b**						
“Sierra” MI	*Cons 7*	SYBR	29.88477135			
“Sierra” MI	*Cons 7*	SYBR	30.33911514	30.19886		
“Sierra” MI	*Cons 7*	SYBR	30.37268639			
“Sierra” I	*Cons 7*	SYBR	28.9278698			
“Sierra” I	*Cons 7*	SYBR	29.36959648	29.31236		
“Sierra” I	*Cons 7*	SYBR	29.63962555			
“Sierra” MI	*Phvul010G025800*	SYBR	27.765		0	1
“Sierra” MI	*Phvul010G025800*	SYBR	26.9359	27.19686	0	1
“Sierra” MI	*Phvul010G025800*	SYBR	26.88967		0	1
“Sierra” I	*Phvul010G025800*	SYBR	23.62484		2.57595	5.96262
“Sierra” I	*Phvul010G025800*	SYBR	23.68967	23.73442	2.57595	5.96262
“Sierra” I	*Phvul010G025800*	SYBR	23.88874		2.57595	5.96262

## Supplementary Materials

Supplementary materials can be found at www.mdpi.com/1422-0067/18/6/1109/s1.
