# Supplementary material for: Marker-Assisted Molecular Profiling, Deletion Mutant Analysis, and RNA-Seq Reveal a Disease Resistance Cluster Associated with Uromyces appendiculatus Infection in Common Bean Phaseolus vulgaris L
Source: Int J Mol Sci. 2017 May 23;18(6):1109. doi: 10.3390/ijms18061109 (PMC5485933; doi:10.3390/ijms18061109)
Supplement: Supplementary file 1 [file ijms-18-01109-s001.pdf]

Supplementary Information

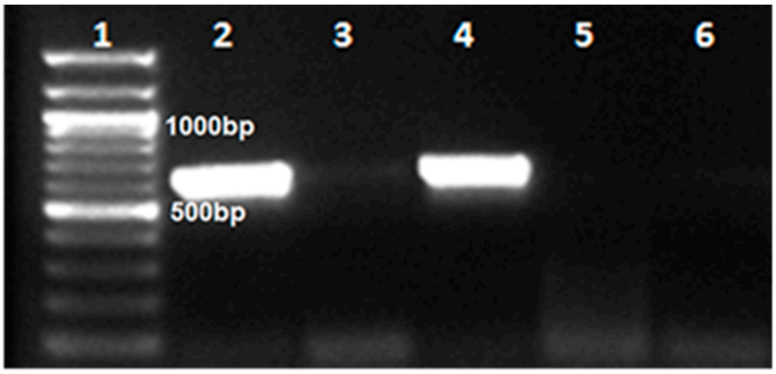

Figure S1. Molecular marker SK14 amplified in "Sierra" and crg genomic samples. This marker does not amplify in "Olathe", ur3-Δ2 or ur3-Δ3

Table S1. Primer sets and amplicon sizes for q-PCR of genes in delineated region

Sequence Name	Bases	Sequence	Amplicon size
010G024900 FWD Set 4	21	CTG AAG GTG GAA CAG TGA GTT	
010G024900 REV Set 4	20	CCT TAC CGG GTT GAG TTT GT	119 bp
010G025000 FWD Set 2	22	ACA GAA GCC ATT CAG GGA TTA G	
010G025000 REV Set 2	20	CTC CAG CAA GTT GGA CAT GA	127 bp
010G025100 FWD Set 1	23	CAG CAA CAG ATA CCA CTC TAC AT	
010G025100 REV Set 1	22	GTG ATG CCT GAG GAA GAC TAA A	105 bp
010G025200 FWD Set 4	20	CGA CAT CTT CTG CCC GTT TA	
010G025200 REV Set 4	19	ATG CCA TGC TCC AGT TCT T	113 bp
010G025300 FWD Set 1	22	GCT GCC AGC GAT AAC ATA TCT A	
010G025300 REV Set 1	20	CTG GAG GCA CAC TGA AAG AA	133 bp
010G025400 FWD Set 4	21	CCG TGT GGA AGA TGT GAT TCT	
010G025400 REV Set 4	20	TTG ACG GTA GAT GGC TTT GG	118 bp
010G025500 FWD Set 1	21	TTG ATG AGG ATG CGG GAT ATG	
010G025500 REV Set 1	20	GTC GCA GCA GTC TTG ATT TG	102 bp
010G025600 FWD Set 2	20	GTT TGA ACC ACG CAG TTA CC	
010G025600 REV Set 2	21	CCG TCC ATT CTC AAA CAC AAC	101 bp
010G025700 FWD Set 1	21	GGG CAG ATG GAA TCC TTA ACA	
010G025700 REV Set 1	21	TCG TGC CAA TCC TTC ATA TCC	126 bp
010G025800 FWD Set 4	20	AAC AGT CCA CGT GGC ATA AG	
010G025800 REV Set 4	20	CTC TGC GGT TCC AAC GAA TA	131 bp
010G025900 FWD Set 1	20	CCA CGC AGG TCC AAT CTA AA	
010G025900 REV Set 1	23	GTG AGA TGT CCA ATC CTC TCT TC	142 bp
010G026000 FWD Set 3	22	GAG GGA ATG ATG CCG AGA ATA C	
010G026000 REV Set 3	20	GCT CTT GAA CAC ACC GAG AA	106 bp
010G026100 FWD Set 3	20	CGT GGC AAC TGA CTG TCT TA	
010G026100 REV Set 3	20	CTT GTC TCC AGC TCC CAA AT	142 bp
010G026200 FWD Set 3	20	GCG TCA TCA GAA GGG TGA TT	
010G026200 REV Set 3	22	CTG ACA TCC CAA CCA CTG ATA C	141 bp
010G026300 FWD Set 3	20	TCC GTC GCA TGT TCG TAA TC	

010G026300 REV Set 3	20	CAG CCT CCC AGA GTG AAT TT	128 bp
010G026400 FWD Set 2	20	GAA ATC ACG GCA ACC GAA AG	
010G026400 REV Set 2	20	TTG TCT CCA CCT CCC AAA TG	146 bp
010G026500 FWD Set 5	21	GGA CTC ATT TGC GTT CAT TGG	
010G026500 REV Set 5	22	TGG TCT TGA CGA TGT GAA AGA G	136 bp

Table S2. All genotypes used in this study with genotype, molecular marker and phenotype information. Molecular marker SK14 primer set amplifies in 'Sierra' and crg genomic DNA. SB1 amplifies in all genomic DNA except for crg.

	Genotype	Markers	Phenotype
'Sierra'	(*Ur-3,Ur-3*/*Crg,Crg*)	SK14/SB1	Resistant to race 53
'Olathe'	(*ur-3,ur-3*/*Crg,Crg*)	SB1	Susceptible to race 53
crg	(*Ur-3,Ur-3*/*crg,crg*)	SK14	Susceptible to race 53
ur3-Δ2	(*ur-3,ur-3*/*Crg,Crg*)	SB1	Susceptible to race 53
ur3-Δ3	(*ur-3,ur-3*/*Crg,Crg*)	SB1	Susceptible to race 53
